# Predicting the Risk of Lyme Disease: Habitat Suitability for *Ixodes scapularis* in the **North Central United States**

**DOI:** 10.3201/eid0803.010166

**Published:** 2002-03

**Authors:** Marta Guerra, Edward Walker, Carl Jones, Susan Paskewitz, M. Roberto Cortinas, Ashley Stancil, Louisa Beck, Matthew Bobo, Uriel Kitron

**Affiliations:** * University of Illinois College of Veterinary Medicine, Urbana, Illinois, USA; † Michigan State University, East Lansing, Michigan, USA; ‡ University of Wisconsin, Madison, Wisconsin, USA; § NASA Ames Research Center, Moffett Field, California

**Keywords:** Lyme disease, *Ixodes scapularis*, *Borrelia burgdorferi*, geographic information systems (GIS), north-central United States, risk map

## Abstract

The distribution and abundance of *Ixodes scapularis* were studied in Wisconsin, northern Illinois, and portions of the Upper Peninsula of Michigan by inspecting small mammals for ticks and by collecting questing ticks at 138 locations in state parks and natural areas. Environmental data were gathered at a local level (i.e., micro and meso levels), and a geographic information system (GIS) was used with several digitized coverages of environmental data to create a habitat profile for each site and a grid map for Wisconsin and Illinois. Results showed that the presence and abundance of *I. scapularis* varied, even when the host population was adequate. Tick presence was positively associated with deciduous, dry to mesic forests and alfisol-type soils of sandy or loam-sand textures overlying sedimentary rock. Tick absence was associated with grasslands, conifer forests, wet to wet/mesic forests, acidic soils of low fertility and a clay soil texture, and Precambrian bedrock. We performed a discriminant analysis to determine environmental differences between positive and negative tick sites and a regression equation to examine the probability of *I. scapularis* presence per grid. Both analyses indicated that soil order and land cover were the dominant contributors to tick presence. We then constructed a risk map indicating suitable habitats within areas where *I. scapularis* is already established. The risk map also shows areas of high probability the tick will become established if introduced. Thus, this risk analysis has both explanatory power and predictive capability.

Lyme disease, the most common vectorborne disease of humans in the United States, is caused by the spirochete *Borrelia burgdorferi* and transmitted by the blacklegged tick *Ixodes scapularis*. The distribution of Lyme disease in the Midwest has been determined largely by mapping the distribution of its vector, *I. Scapularis,* which was first detected in northwestern Wisconsin in the late 1960s (*2*). Its range then expanded southward and eastward (*3*–*6*). Even though an isolated established population was discovered in northeastern Wisconsin in Marinette County (*7*), *I. scapularis* does not appear to have become established in several counties in northeastern Wisconsin. This area is heavily populated with white-tailed deer (*Odocoileus virginianus*) and white-footed mice (*Peromyscus leucopus*) (*8*), which serve as hosts for *I. scapularis* (*1*). Since host densities do not appear to be a limiting factor for the tick population (*9*), the physical environment, both at the macro and micro levels, may affect the tick’s ability to survive in this habitat. Moreover, even if establishment is successful, environmental factors may limit tick population densities.

In northwestern Illinois, well-established *I. scapularis* populations were found along the Rock River in Ogle County and in Rock Island and Lee counties since the late 1980s (*10*–*14*). Through the early 1990s, Jo Daviess County was the only positive area along the Wisconsin border, and Putnam County was the only positive along the Illinois River. In southern Illinois, no blacklegged ticks were found among white-tailed deer in a survey conducted from 1980 to 1983 (*15*). Northern Illinois also maintains populations of white-tailed deer and white-footed mice (*8*), although a large proportion of land is used for agriculture (*16*).

The phenology of *I. scapularis* has been studied in Michigan (*17*), Wisconsin (*18*), and Illinois (*19*). In the Midwest, adults have both a longer activity period as well as higher peak densities in the spring than in the fall.

Studies of habitat preferences of *I. scapularis*, which have been conducted at various spatial scales (*20*–*22*), found environmental factors that are associated with vector and host distribution and densities. *I. scapularis* presence has been correlated with sandy soils (*23*,*24*) and wooded vegetation (*25*–*28*). At the macro level, environmental risk factors for Lyme disease have been determined using satellite, climatological, and ecological data to characterize the habitat of the vector tick using geographic information systems (GIS), both in Europe (*29*–*33*) and the United States (*22*–*24*,*34*–*36*).

The purpose of this study was to determine the distribution of *I. scapularis* in the upper Midwest based on data from Wisconsin, northern Illinois, and the Upper Peninsula of Michigan, and to explain the environmental factors that facilitate or inhibit the establishment of *I. scapularis*. Since host abundance is not a limiting factor for the maintenance of tick populations in this area, survival of *I. scapularis* may depend on a combination of several environmental risk factors, resulting in a patchy, discontinuous distribution of this vector. We propose a hierarchic interpretation, starting from the bedrock geology through glacial history and climate patterns, to explain the topography, soil, and vegetation patterns that may directly affect tick survival. By characterizing the habitat preferences of *I. scapularis* using digitized databases (some derived from satellite imagery) and field data integrated into a GIS, the distribution of Lyme disease and other diseases transmitted by the blacklegged tick can be predicted, and the risk of transmission to the human population can be assessed.

## Methods

### Site Selection

In Wisconsin, a statewide survey of parks and forests was conducted to determine the presence of *I. scapularis*. Sites were selected to represent each region in the state, with 28 of 59 states parks and forests selected. In Michigan, three sites were selected in Menominee County, where *I. scapularis* had previously been identified (*7*). In Illinois, paired positive and negative sites were sampled in Ogle and Rock Island counties, and additional sites were sampled along the Illinois River. Data are presented separately for each collection site.

### Tick Collection

Tick collection was conducted at a total of 138 sites in July and September-October 1996, June 1997, and May-June 1998. The most comprehensive trips were made June 14-26, 1997, and May 27 through June 3, 1998, in the southern part of the study region. In several natural areas, more than one site was dragged, and results for each site were considered separately.

Questing *I. scapularis* ticks were collected in two ways: 1) by dragging a 1-m^2^ white flannel cloth through vegetation for a total of at least two hours at each site (timed dragging), or 2) by dragging 1000 m on a grid (distance dragging). Timed dragging was conducted by teams of 4 or 5 persons, with each person dragging for 30 minutes. Distance dragging was also conducted by teams of 4 or 5 persons, which required an estimated 2 to 2.5 hours per grid. Thus, each site was dragged for a total of 2 or 2.5 hours per visit. All calculations of tick numbers are per 1 drag-hour.

Nymphs and adults were maintained alive in plastic vials with moistened cotton balls on ice for *B. burgdorferi* culture. Larvae were placed in vials containing 70% ethanol for later identification.

### Vertebrate Collection

Small mammals were trapped overnight during July and October 1996, June 1997, and June 1998 at 13 selected sites in Wisconsin, and at all the Michigan and Illinois sites. Sherman live traps (H.B. Sherman Traps, Inc, Tallahassee, FL) were placed approximately 10 meters apart and baited with bread and peanut butter. Approximately 35 to 50 traps were placed per site, and 0 to 15 mice and 0 to 7 chipmunks were trapped at each site. White-footed mice and chipmunks were anesthetized with the inhalant anesthetic methoxyflourane (Shering-Plough, Inc., Madison, NJ), examined for ticks, and ear-tagged, and their sex and weight were recorded (LACAC animal use protocol # 99099)*.* Ticks were removed and placed in vials containing 70% alcohol for identification.

### Site Classification

For each site, the average number of each stage of the deer tick was calculated per hour of dragging. The number of ticks per dragging hour is based on an average of all drags. There was no situation where all or most ticks were found on one drag. The average number of larvae and nymphs was determined per small mammal captured. These data were not pooled with the dragging data because animals were not trapped at all sites.

A site was classified as negative (0) if *I. scapularis* was never found on vegetation or small mammal hosts. There was no case where ticks were found only on small mammals but not on drags. A site was rated 1 if only one stage of the tick was found, regardless of the quantity. A rating of 2 was given if all stages of the tick were found at low density (<10 larvae, <4 nymphs, <2 adults), and a rating of 3 indicated all stages were found at higher density.

We considered several types of classification, including calculating each stage separately and each collection trip separately. Although repeat visits increase the chance that a site will be classified as positive for the presence of ticks, there were no sites where more than one stage was found in only one visit. The finding of only one stage, however, may indicate accidental introduction without establishment. We selected a very conservative and coarse classification to account for the limitations of such an extensive field survey and to allow for differences in weather conditions, time of day, and other variables.

#### Soil Data

After removing the layer of leaf litter, soil samples were collected at each site from the uppermost 6 inches of topsoil. Data on predominant vegetation, leaf litter thickness, slope, and compass direction were also recorded at each site. Particle size analysis was performed on 10 gm samples of soil (*37*). The pH and the percentages of sand, silt, and clay were measured for each sample, and the soil texture class was determined from a combination of these percentages. The percentages and classes were compared with site positivity using Spearman rank correlation.

#### Forest Moisture Index

The classification of forest type was derived from the predominant trees at each site. The number of mature trees (>4 inches in diameter) were counted within a 50-m^2^ grid at each site and identified according to species (*38*). The most common species were used to classify the forests via a moisture index (*38*). The sites were divided into five categories: dry, dry/mesic, mesic, wet/mesic, and wet.

### Georeferenced Databases

#### Data Sources

Geographic coordinates of sites were determined by using a Trimble Geoexplorer (Trimble Navigation, Ltd., Sunnyvale, CA) global positioning system (GPS) and exported by using the Trimble Pathfinder software into ARC/INFO and ArcView GIS (ESRI, Redlands, CA). The generated georeferenced database was overlaid on digitized state coverages of environmental data. Land cover and elevation data for Wisconsin were obtained from WISCLAND/GAP (University of Wisconsin and Wisconsin Department of Natural Resources, Madison, WI) at a scale of 1:40,000. WISCLAND/Upper Midwest GAP analysis created land cover classifications based on Landsat Thematic Mapper (TM) data and stratification of the satellite imagery with a hierarchic classification system into wetlands, urban areas, and upland areas. For Illinois, land cover, elevation, and quaternary geologic data were obtained from the Illinois GIS (Department of Natural Resources, 1996, Springfield, IL) at a scale of 1:500,000. Bedrock geology data were obtained from the Digital Geologic Map and Mineral Deposits of Minnesota, Wisconsin, and Michigan (U.S. Geological Survey, Reston, VA) at a scale of 1:1,000,000 for Wisconsin and Michigan, and from the Illinois GIS at a scale of 1:500,000. Soil data, including order, texture, drainage, and quaternary geology, were obtained from STATSGO (U.S. Department of Agriculture, Washington, DC) with a resolution of 2.5 km^2^.

Climate data, gathered by the weather station closest to each site, were obtained from the National Oceanographic and Atmospheric Administration (National Climate Data Center, Asheville, NC). Variables included yearly and seasonal precipitation. Landsat TM satellite images were obtained for the entire study area from summer and fall of 1989 through 1993. For each site, average values of TM bands 3 (red), 4 (near-infrared), 5 (mid-infrared), and the normalized difference vegetational index (NDVI) were calculated for the surrounding 3x3 (0.01 km^2^), 10x10 (0.1 km^2^), and 30x30 (0.9 km^2^) pixels. Indices of greenness, brightness, and wetness were obtained through the tasseled-cap transformation (*39*). Brightness is a measure of reflectance and is correlated to the texture and moisture content of soils, while greenness is a measure of the density of green vegetation present. Wetness is a measure of moisture in soils and vegetation. These remote sensing indices were treated as interval-level data and were associated with tick abundance at each site.

#### Environmental Variables

Land cover data were grouped into five ordinal categories: agriculture, grasslands, coniferous forest (in which >75% of trees maintain leaves all year), mixed forest (neither deciduous nor coniferous species make up >75% of land cover), and deciduous forest (at least 75% of trees shed foliage simultaneously in response to seasonal change).

Bedrock geology was classified as Precambrian, which consists of volcanic and metamorphic rocks, and sedimentary deposits from the Silurian, Ordovician, and Devonian eras (*40*). Quaternary geology information was obtained from the USDA Forest Service North Central Research Station (General Technical Report NC-178). Categories were classified as outwash plains and pitted outwash, lake plain, till plain, ground moraine, loess, and plateau.

Soil orders are defined by amount of organic matter present, pH, and the type of vegetation growing on the soil (*40*). In Wisconsin and northern Illinois, 8 of 12 soil orders are represented: mollisols (present under prairie), alfisols (deciduous forests), spodosols (coniferous forests), entisols and inceptisols (both of which are associated with poorly developed soils),histosols (peat and muck), and vertisols and paleosols (which represented <1% of the area). These orders were classified into ordinal categories based on increased fertility and decreased acidity: 0 = histosol and spodosol, 1 = entisol, 2 = inceptisol, 3 = mollisol, and 4 = alfisol.

Soil texture (*40*) was divided into seven groups in order of increasing particle size, ranging from clay (<2 mm) through silt (2 to 50 mm) to sand (0.05 to 2.0 mm). Drainage was divided accordingly into seven categories (STATSGO, Washington, DC), from very poorly drained to well drained. Excessively drained soils were ranked as 0 since they are too dry to support a biotic environment (*40*).

For each site, yearly and seasonal rainfall averages and average snowfall per year were obtained from the weather station (NOAA) nearest each site. Elevation ranged from 495 m in northern Wisconsin to 197 m in western Illinois. Precipitation, elevation, and remote sensing indices were treated as interval-level data.

### Statistical Analysis

All analyses were performed by using SPSS software (SPSS, Chicago, IL). Soil texture classifications of samples from the sites were compared with those listed in STATSGO, the soils database (STATSGO, Washington, DC, and Spearman rank correlation was used to assess correlations between field data and data from the GIS. Univariate analysis was initially performed by using chi square contingency tables to determine significant associations between site positivity and environmental variables coded as previously described. Discriminant analysis was performed by using only the significant (p<0.25) environmental variables from the univariate analysis (*41*). A linear discriminant function was obtained from the combination of variables that best characterized the differences between the groups. A stepwise approach was used to enter variables one at a time until the discriminating power between tick abundance categories ceased to improve. Analyses were performed by grouping the outcome variables into positive or negative sites and into the four abundance categories described previously.

As mentioned, since a site classified as category 1 (finding only one stage of the tick) could result from introduction into an unsuitable habitat, categories 0 and 1 were combined for additional analysis. Only 112 sites were used in the analysis, with no more than three sites included per natural area where multiple sites were sampled. The resulting classification functions were then used to predict tick abundance categories and assess how well the functions discriminated. Separate discriminant analyses were performed by using the seven indices obtained from the remote sensing data at three spatial scales and the precipitation data.

Logistic regression analysis was performed by using the primary environmental factors as independent variables and the positive and negative sites as outcome variables. Forest moisture index was excluded from the model because this variable was not available as digitized geographic coverage.

To develop a risk map for Lyme disease in the area studied, a grid was created encompassing the states of Wisconsin and Illinois with a resolution of 2.5 km^2^ per cell. The grid was overlaid with the selected coverages by using ARC/INFO and ArcView GIS(ESRI, Redlands, CA), and data values corresponding to each layer were assigned to each cell. The Summarize Zones procedure from the ArcView Analysis Menu was used to calculate summary attributes for features by using a grid scheme that divided the entire study area into 2.5-km^2^ cells. Each cell was assigned a value for each layer included in the logistic regression based on the most common category. The logistic equation was then used to generate the probability of the presence of *I. scapularis* within each 2.5-km^2^ cell of the grid map. The map was generated with probabilities divided into quartiles and deciles.

## Results

**Figure 1:**
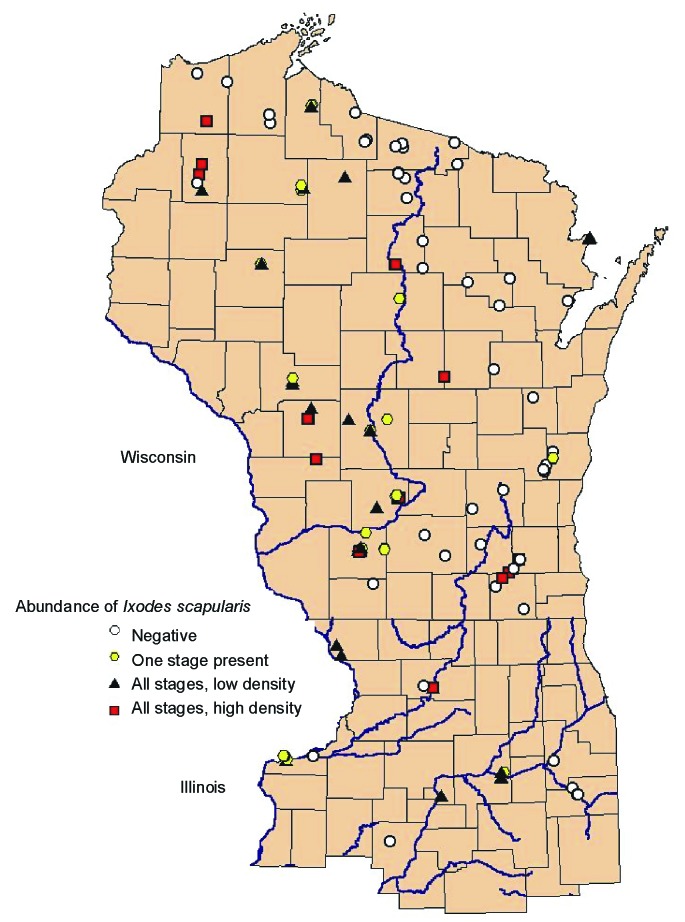
Geographic distribution of study sites ranked by abundance of *Ixodes scapularis* in Wisconsin, northern Illinois, and Menominee County in Michigan.

The locations of the 138 sites that were sampled in Wisconsin, Illinois, and Michigan are shown in Figure 1. Among the four categories, 56 sites were classified as negative, 24 were ranked as 1, 32 as 2, and 26 as 3. Most negative sites were in northeastern Wisconsin. In the southeastern part of Wisconsin, sites were negative except those situated in the Kettle Moraine State Forests (Sheboygan, Fond du Lac, Jefferson, Walworth, and Waukesha counties), which are located on the terminal glacial moraines. Negative sites in Illinois were at Blackhawk Nature Preserve (Rock Island County), located in a suburban area, and White Pines State Park (Ogle County), which has large stands of secondary growth pine forest. In Wisconsin, positive high-density sites were found in the southwestern driftless area and in the central sandy uplands, as well as in the well-recognized northwest part of the state (and across the state line into Minnesota).

In Michigan, where only a small area of the Upper Peninsula was sampled, all sites had very dense tick populations, except for a site that was classified as excessively drained (>99% sand). The sites classified in the other two abundance categories (1 and 2) did not appear to cluster in any areas. In Illinois, the two parks that have been infested for at least a decade, Castle Rock State Park (Ogle County) and Loud Thunder Forest Preserve (Rock Island County), were classified as having dense tick populations, with lower populations in some sites along the Illinois River.

**Figure 2:**
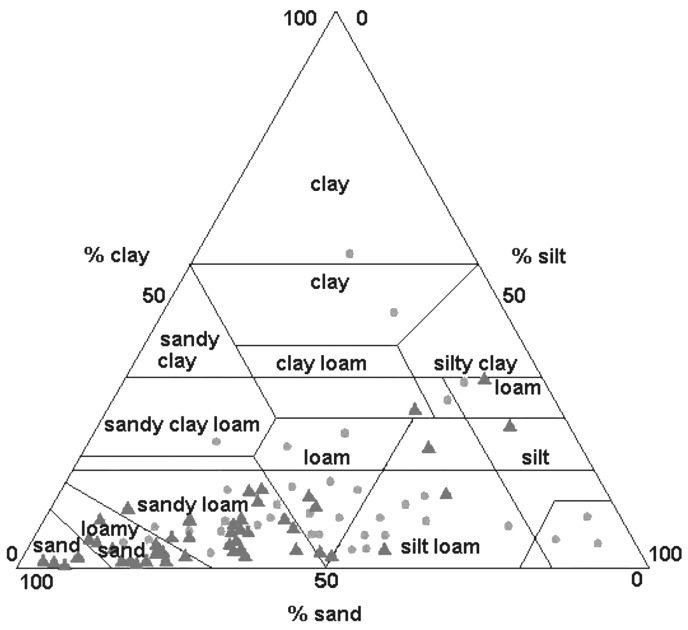
Soil particle size analysis of samples from positive and negative sites. Soil texture is expressed as the sum of percent sand, silt, and clay.

Particle size analysis, which is a function of the proportions of sand, silt, and clay, was performed at 82 sites (Figure 2). The positive sites were clustered in the sand/loamy sand texture classes. Individual percentages of sand, silt, and clay per sample were not correlated with tick abundance; however, texture class, which is a combination of these three percentages, correlated significantly (r=0.42, p<0.05) with greater tick densities found in soils with a greater proportion of sand. The soil texture class of samples determined from the soil analysis correlated significantly (r=0.46, p<0.001) with the soil texture class of each site as obtained from the STATSGO database.

**Figure 3:**
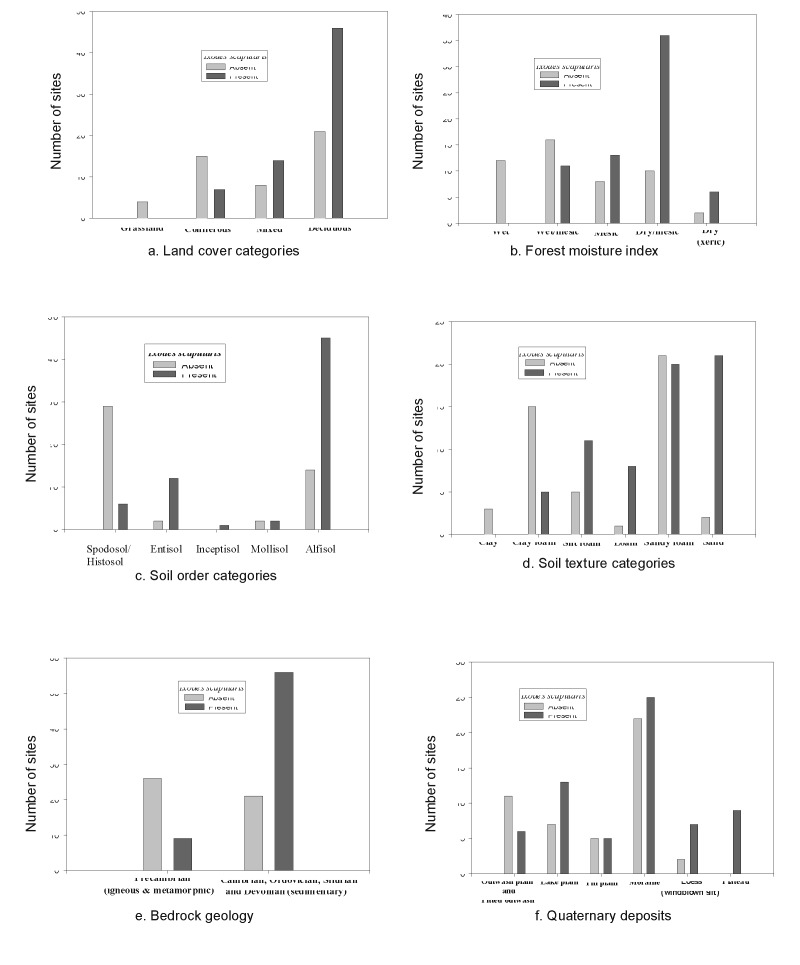
Categories of environmental variables and number of positive and negative sites.

**Figure 4:**
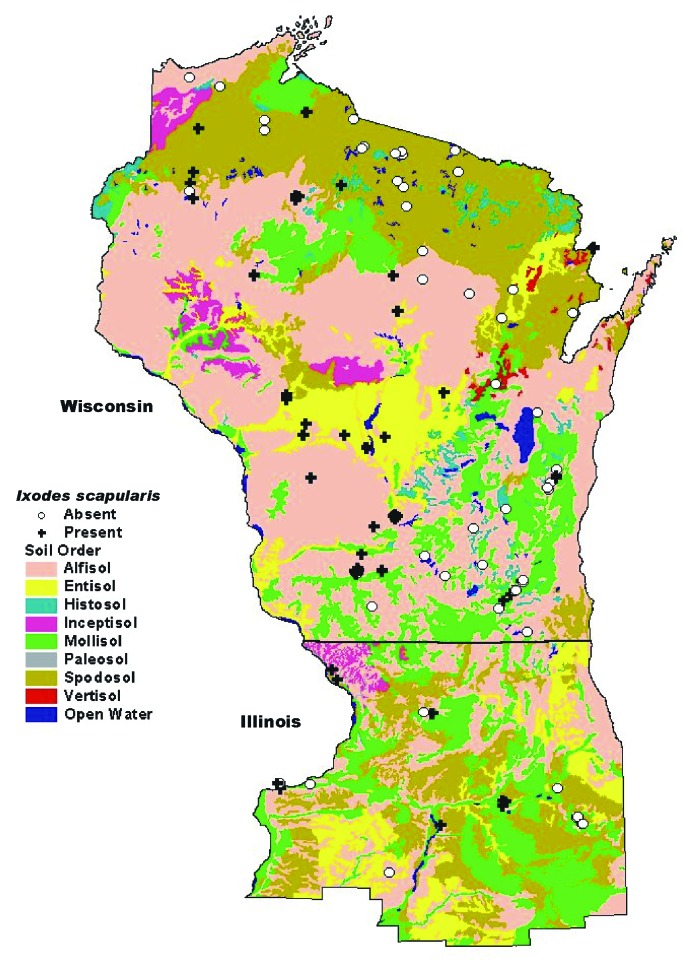
Map of soil orders in Wisconsin and northern Illinois, overlaid with tick study sites.

The univariate analysis detected significant associations (p<0.25) between tick presence and land cover, soil order, bedrock geology, quaternary geology, soil texture, forest type, spring, summer, fall and winter precipitation, snowfall, and elevation (Figure 3). The results of the discriminant analysis are listed in Table 1. When negative and positive sites were contrasted, the variables forest type, soil order, land cover, soil texture and bedrock were significant. Tick presence was positively associated with deciduous (Figure 3a), dry/mesic and dry forests (Figure 3b), fertile soils such as alfisols (Figures 3c, 4), sand and loamy/sand soil texture (Figures 2, 3d), and sedimentary bedrock (Figure 3f). There was a negative association with grasslands and conifer forests (Figure 3a), wet and wet/mesic forests (Figure 3b), acidic soils such as spodosols (Figure 3c), clay soil texture (Figure 3d), and Precambrian bedrock (Figure 3e). Elevation was not an important discriminator in the model, nor was Quaternary geology (Figure 3f) important even though sites located on the plateaus and loess-covered areas were all positive. However, the distribution of the sites among the categories of Quaternary deposits was skewed because a large proportion of the state parks were located on terminal glacial moraines. The discriminate model was able to correctly classify 85.7% of the sites. The canonical correlation coefficient was 0.69, and the eigenvalue was close to 1 (0.92), indicative of a strong discriminant function. When the single stage category (*1*) was included with the negative group, only two variables, forest type and soil order, were significant. Most of the sites (78.6%) were still correctly classified; however, the eigenvalue decreased to 0.43. These same variables were significant when all the groups were considered separately; but the model only correctly classified 51.8% of the sites. Even though only 4/33 in the negative group were misclassified, there was very poor discrimination among the tick positive groups. No significant variables resulted from the discriminant analysis performed using the satellite data. Since all sites were located in forested areas, TM imagery may not have been able to discriminate well among suitable and unsuitable forested habitats. The precipitation variable was also not a significant discriminator between positive and negative sites in the model.

**Table 1:** Significant environmental variables to determine favorable habitat for *Ixodes scapularis* using discriminant analysis

Groups (sample size)							
	0 vs. 1,2,3 (47 vs. 65)		0,1 vs. 2,3 (63 vs. 69)		0 vs. 1 vs. 2 vs. 3 (47 vs. 16 vs. 24 vs. 25)		
Variable	Wilk’s lambda	Disc F(x)	Wilk’s lambda	Disc F(x)	Wilk’s lambda	Disc F(x)1	Disc F(x)2
Forest type	0.784	0.552	0.789	0.789	0.754	0.665	-0.747
Soil order	0.618	0.521	0.699	0.542	0.569	0.633	0.774
Land cover	0.586	0.387					
Soil texture	0.564	0.381					
Bedrock	0.525	0.518					
Eigenvalue	0.904		0.431		0.681		0.045
Canonical correlation coefficient	0.689		0.549		0.636		0.207
% correctly classified	85.7		78.6		51.8		

**Figure 5:**
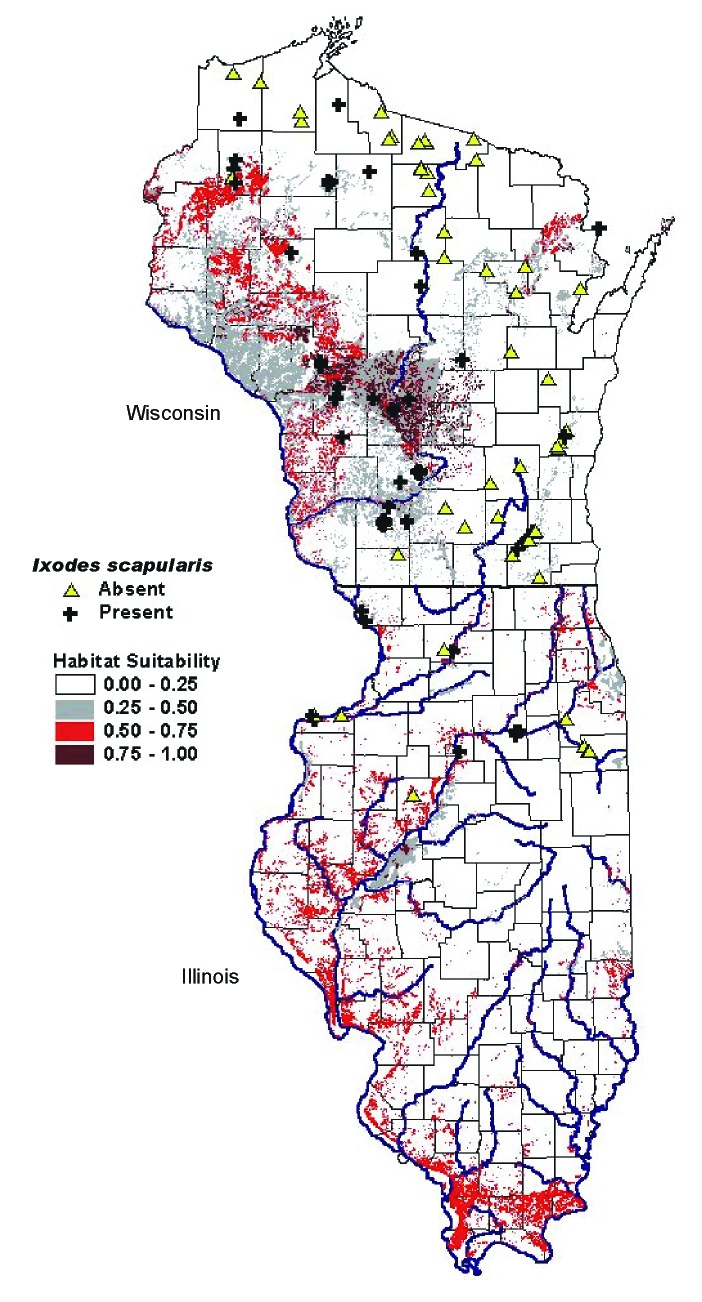
Predictive risk map of habitat suitability for *Ixodes scapularis* in Wisconsin and Illinois.

The results of the logistic regression analysis were in agreement with the discriminant analysis model in the positive versus negative group as seen in Table 2. The same variables were significant (p<0.05), and the model correctly classified 83.9% of the sites. The predictive risk map generated from the logistic regression model is shown in Figure 5. The higher probabilities indicate increased suitability of habitat for *I. scapularis.* In Wisconsin, the areas of moderate suitability (26%-40%) are located in the western half of the state. Patchy areas of higher probability (60%-100%) are found in the central and northern portion (Juneau, Adams, Waushara, and Marquette counties.) and along the border with Minnesota (Vernon and Crawford counties). In Illinois, the positive sites that were sampled corresponded to areas of increased suitability (60%-100%). Castle Rock State Park, where the highest tick densities are found, had a 90%-100% probability of suitable habitat. The areas bordering the Illinois River appear to be adequate habitat for *I. scapularis*, especially on the western side. Shawnee National Forest in the extreme southern portion of the state also appears to have a high probability (60%-80%), even though *I. scapularis* populations have not been detected (*42*).

**Table 2:** Significant environmental variables in the logistic regression model.

					95% Confidence interval
					

Variable	Beta	S.E.	P value	Odds ratio	Lower Upper	
Land cover	0.85	0.40	0.03	2.36	1.08	5.15
Soil order	0.42	0.18	0.02	1.52	1.07	2.16
Bedrock	1.78	0.73	0.01	5.94	1.42	24.78
Soil texture	0.76	0.26	0.004	2.13	1.27	3.57
Constant	-9.06	1.95				

## Discussion

*Ixodes scapularis* may be introduced into new areas by several routes. Adult *I. scapularis* are carried into new areas primarily by deer (*43*), which are capable of ranging over wide areas, especially along riparian corridors. However, infected adult ticks have limited potential for spreading Lyme disease since transovarial transmission of *B. burgdorferi* is rare. Small mammals are efficient disease reservoirs, and juveniles tend to disperse during the spring and summer when tick larvae and nymphs are questing. However, the potential for long-range dispersal of Lyme disease by rodents is limited, since they occupy much smaller home ranges than deer (*44*). Birds have a high potential for introducing infected immature stages of *I. scapularis* into distant areas (*45*–*47*), especially during spring and fall migration.

To become successfully established in a new area, *I. scapularis* requires available hosts for feeding, which is not a limiting factor in our study area, and a suitable habitat for questing, molting, diapause, and oviposition. The vegetation, soil, topography, and climate are interrelated, and extremes of any one factor may adversely affect the tick’s ability to survive.

The environmental characteristics vary throughout the two states, and certain combinations may determine whether introduced *I. scapularis* populations can become established. Tick abundance is an indicator of the suitability of environmental conditions for reproduction and survival. Finding only one stage of the tick may indicate either a poor microenvironment or a recent introduction. Finding all three stages at one site strongly suggests that a population has become established. A less than optimal habitat may account for low density in an established *I. scapularis* population, or it may indicate a recent introduction. Errors in classification may occur in an extensive field survey, as reported here, and a dynamic situation (i.e., the process of invasion of a new site) may mask the occurrence of some positive or potentially positive sites. By including a large number of sites and conducting repeat visits, we have tried to minimize such confounding effects.

Environmental factors such as bedrock geology, quaternary deposits, soils, vegetation, and climate influence each other directly and indirectly to create unique habitats. This is why we included risk factors that are not necessarily independent in a model that is most unbiased. The soil orders in the region (Figure 4) are influenced by the type of underlying bedrock and by quaternary deposits. The soils, in turn, influence the type of vegetation overlying them. Soil texture is the component of soil that influences the extent of drainage. The soil texture classes are independent of soil order and are usually a function of the degree of soil weathering and the parent material (bedrock or quaternary deposit). The tree composition of a forest is determined by a moisture gradient involving soil aeration, soil nutrient supply, and microclimatic features (*38*), and this gradient functions as a continuum. The forest types classified as dry and dry/mesic have oaks and jack pines as the dominant species that prefer well-drained, sandy soils. Oak forests also have a dense canopy layer that provides protection for the underlying vegetation. Wet and wet/mesic forests are composed of trees that have a high tolerance for very moist soils. The factors interacting at the microclimatic level within the topsoil and leaf litter appear to have an important influence on tick survival. Excessively moist conditions at the soil level were negatively associated with the establishment of *I. scapularis*. Soil texture, in addition to the topography, determines the extent of drainage, and the level of moisture of the ground layer, regardless of the amount of precipitation. However, given the effect of weather on tick abundance (*19*), associations between tick presence and amount of yearly precipitation or snowfall need to be analyzed further.

Our findings suggest that abiotic factors play a major role in determining whether populations of *I. scapularis* can become established in an environment. Precambrian bedrock of volcanic origin results in the formation of acidic soils that are found mainly under coniferous forests, the forest type least likely to support tick populations. Soils containing increased acidity (spodosols) and a high proportion of clay that can retain excess moisture (*48*) were also more frequently present in negative sites. Excessive moisture in the soil may be deleterious to tick survival since they overwinter in the topsoil and leaf litter. It may also enhance the growth of organisms, such as fungi and entomophagous nematodes, which may have adverse effects on the tick population (*49*). Leaf litter is a necessary component for the survival of immature stages of *I. scapularis* (*50*). However, the type and quantity may determine the densities of ticks in a specific habitat. Tick densities were highest in forests dominated by oak, followed by maple, and lowest in coniferous forests that produce minimal amounts of leaf litter (*38*). Tick densities were also highest in areas with underlying sedimentary bedrock, which is associated with alfisol and mollisol soil orders and soil textures of increased particle size (*38*).

The statistically significant risk factors derived from the logistic regression analysis were in agreement with those obtained from the discriminant analysis, and allowed us to quantify and predict the environmental risk for the presence of *I. scapularis*. Several environmental factors must be evaluated simultaneously to assess the combination of factors required for successful establishment. Determining the environmental factors that limit survival can facilitate the development of measures for the control of the tick in the environment.

Using a GIS, we generated a risk map (Figure 5) to predict the presence of the tick vector*, I. scapularis*. The areas of suitable habitat for *I. scapularis* in Wisconsin corresponded to areas of increased incidence of human Lyme disease and known areas of tick endemicity. The extensive area of suitable habitat in the western portion of the state can explain the rapid expansion of the tick from the original northwestern focus to the southwestern portion of the state (*2*–*5*). While initial studies of tick distribution (*3*) and human granulocytic ehrlichiosis (*51*) point to the risk of tick-borne disease transmission in Northwest Wisconsin, our study points also to the sandy barrens of Central Wisconsin as most suitable habitats. Indeed, the highest numbers of ticks were collected in Council Ground State Park (Lincoln County), Fort McCoy (Monroe County), Hartman Creek State Park (Waupaca County), and Wildcat Mountain State Park (Vernon County), as well as in sites in the long-recognized Spooner area (Washburn County). Further, the highest prevalence of canine seropositivity to *B. burgdorferi* in northern Illinois and Wisconsin was found in dogs in the west-central counties of Wisconsin (*52*). Based on the risk map, most of the north-central and northeastern portions of Wisconsin have a <25% probability for tick presence. These are areas where our sampled sites were consistently negative for *I. scapularis*. In the eastern half of the state, the main areas of increased suitability were along the glacial terminal moraines, which is where the positive sites in the Kettle Moraine State Forest were located. There was also a higher probability in the northeastern corner of the state bordering Menominee County, Michigan, where positive sites were located.

In Illinois, areas of increased suitability corresponded to the same areas where the positive sites were located in Ogle, Rock Island, and Jo Daviess counties. The risk map indicated there is adequate habitat for *I. scapularis* populations to become established along the Illinois River, as well as the Mississippi River. However, in Illinois, tick populations may be limited to river corridors since extensive areas are used for agriculture. Where forested habitat is sparse, tick establishment may be restricted, even though geologic and soil factors are favorable. In southern Illinois, where climatic conditions may differ and other reservoir hosts may be present, the inclusion of additional parameters to the model may result in reduced risk probabilities. In contrast, the risk factor model and predictive map may be valid for other north-central areas that have similar environmental characteristics, particularly in parts of Minnesota, Michigan, northern Indiana, and Ohio. The model may be applied to other areas of the United States by using local geographic coverages.

In conclusion, this model can be used to help determine the risk of acquiring Lyme disease and other diseases transmitted by *I. scapularis* by predicting which locations may be currently infested with the tick. It can also be used to assess whether habitats that are currently nonendemic for *I. scapularis* would have the necessary combination of environmental factors to allow new populations of *I. scapularis* to become established. The model can thus be continuously refined based on findings from new areas. The risk of Lyme disease transmission could be predicted in areas capable of sustaining *I. scapularis* populations if ticks harboring *B. burgdorferi* are introduced by migrating deer or birds. The results obtained from these field studies can also form the basis for controlled experimental studies under field and laboratory conditions to further elucidate the preferred microenvironment of *I. scapularis*.
